# The mechanism of NLRP3 inflammasome activation and its pharmacological inhibitors

**DOI:** 10.3389/fimmu.2022.1109938

**Published:** 2023-01-18

**Authors:** Xiaoyan Zhan, Qiang Li, Guang Xu, Xiaohe Xiao, Zhaofang Bai

**Affiliations:** ^1^ Department of Hepatology, Fifth Medical Center of Chinese PLA General Hospital, Beijing, China; ^2^ China Military Institute of Chinese Materia, Fifth Medical Center of Chinese PLA General Hospital, Beijing, China

**Keywords:** pattern recognition receptor, NLRP3, inflammasome, inflammatory diseases, pharmacological inhibitors

## Abstract

NLRP3 (NOD-, LRR-, and pyrin domain-containing protein 3) is a cytosolic pattern recognition receptor (PRR) that recognizes multiple pathogen-associated molecular patterns (PAMPs) and damage-associated molecular patterns (DAMPs). Once activated, NLRP3 initiates the inflammasome assembly together with the adaptor ASC and the effector caspase-1, leading to caspase-1 activation and subsequent cleavage of IL-1β and IL-18. Aberrant NLRP3 inflammasome activation is linked with the pathogenesis of multiple inflammatory diseases, such as cryopyrin­associated periodic syndromes, type 2 diabetes, non-alcoholic steatohepatitis, gout, and neurodegenerative diseases. Thus, NLRP3 is an important therapeutic target, and researchers are putting a lot of effort into developing its inhibitors. The review summarizes the latest advances in the mechanism of NLRP3 inflammasome activation and its pharmacological inhibitors.

## 1 Introduction

Inflammasomes are a class of complexes made up of cytosolic proteins, mediating inflammatory response to pathogen infection and damage to the host (1). Inflammasome is usually composed of a pattern-recognition receptor (PRR), an adaptor known as ASC, and the effector caspase-1.Once activated by an inflammatory ligand, inflammasomes trigger the auto-processing of caspase-1 into the catalytically active caspase-1, which mediates the cleavage of pro-IL-1β and pro-IL-18 (2, 3) and induces pyroptosis (4).

There are several PRRs that could form inflammasomes, including NLRP1, NLRP3, AIM2, NLRC4, and IFI16 (5–7). NLRP3 inflammasome is the best characterized one. NLRP3-activating mutations have been reported to cause cryopyrin­associated periodic syndromes (CAPS) (8). Moreover, a variety of sterile danger signals in tissues, which induce chronic inflammation also activate NLRP3, so NLRP3 inflammasome is central to the pathogenesis of many chronic inflammatory diseases such as gout, type 2 diabetes, non-alcoholic steatohepatitis (NASH), atherosclerosis, and Alzheimer’s disease (AD) (9–13). Consequently, NLRP3 is regarded as an essential target for pharmacological intervention to treat related inflammatory disorders.

To date, various pharmacological inhibitors of NLRP3 inflammasome have been identified and exhibit therapeutic effects in chronic inflammatory diseases. In this review, we introduce the latest findings in the mechanism of NLRP3 inflammasome activation and the reported inhibitors of NLRP3 inflammasome, which may benefit the treatment of NLRP3 inflammasome-driven diseases.

## 2 Mechanism of NLRP3 inflammasome activation

**Figure 1:**
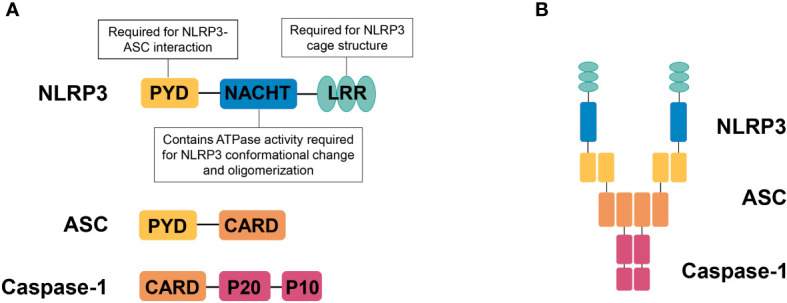
NLRP3 inflammasome. **(A)** Domain organization of NLRP3, ASC, and caspase-1. NLRP3 contains an amino­terminal PYRIN (PYD) domain, a nucleotide­binding NACHT domain, and a carboxy­terminal leucine­rich repeat (LRR) domain. The PYD domain is important for recruitment of ASC; the NACHT domain contains ATPase activity required for NLRP3 conformational change and oligomerization. The LRR domain is important for the formation of the NLRP3 cage, which is required to disperse the trans-Golgi network at the early stage of NLRP3 inflammasome activation. **(B)** NLRP3 inflammasome assembly. Upon activation, NLRP3 recruits ASC *via* PYD-PYD interactions and ASC recruits caspase-1 *via* CARD-CARD interactions.

NLRP3 is a member of NLR family, containing an amino­terminal PYRIN (PYD) domain, a nucleotide-binding NACHT domain, and a carboxy­terminal leucine­rich repeat (LRR) domain (**Figure 1A**). The PYD domain is important for the recruitment of ASC; the NACHT domain contains ATPase activity required for NLRP3 conformational change and oligomerization. The role of LRR domain in NLRP3 activation is controversial, but recent structural studies have shown that the LRR domain is important for the formation of the NLRP3 cage, which is required to disperse the trans-Golgi network at the early stage of NLRP3 inflammasome activation (14). Upon responding to stimuli that cause loss of autoinhibition (15), NLRP3 oligomerizes through interactions between NACHT domains, then recruiting ASC *via* PYD-PYD interactions, ASC self-associates into helical filaments, which further form a single macromolecular focus-termed ASC speck (16–18). Assembled ASC recruits caspase-1 and enables self-cleavage and activation of caspase-1 (**Figure 1B**).

NIMA-related kinase 7 (NEK7) has been identified as a scaffolding protein for NLRP3 activation (19–21). NEK7 is a member of NIMA (“never in mitosis gene a”)–related serine-threonine kinase family and important for mitosis, consisting of a catalytic domain and a short N-terminal domain. Both the NACHT and LRR of NLRP3 are involved in the association with NEK7. NLRP3-NEK7 interaction requires the catalytic domain of NEK7 but not impaired by mutations that abolish the catalytic activity of NEK7, showing that NEK7 binds NLRP3 independently of its kinase activity (19). Meanwhile, NEK7 is dispensable for NLRC4 or AIM2 inflammasome activation, demonstrating it is a specifically essential component of NLRP3 inflammasome (19). The specific structural mechanism of NEK7-licensed NLRP3 inflammasome activation has been revealed by Sharif and colleagues (22). Recently, Le Xiao et al. have reported the cryo-EM structures of the active NLRP3 inflammasome disk; it is proposed that NEK7 breaks the inactive NLRP3 cage to transform NLRP3 into the active NLRP3 inflammasome disk (23).

### 2.1 Canonical NLRP3 inflammasome activation

**Figure 2:**
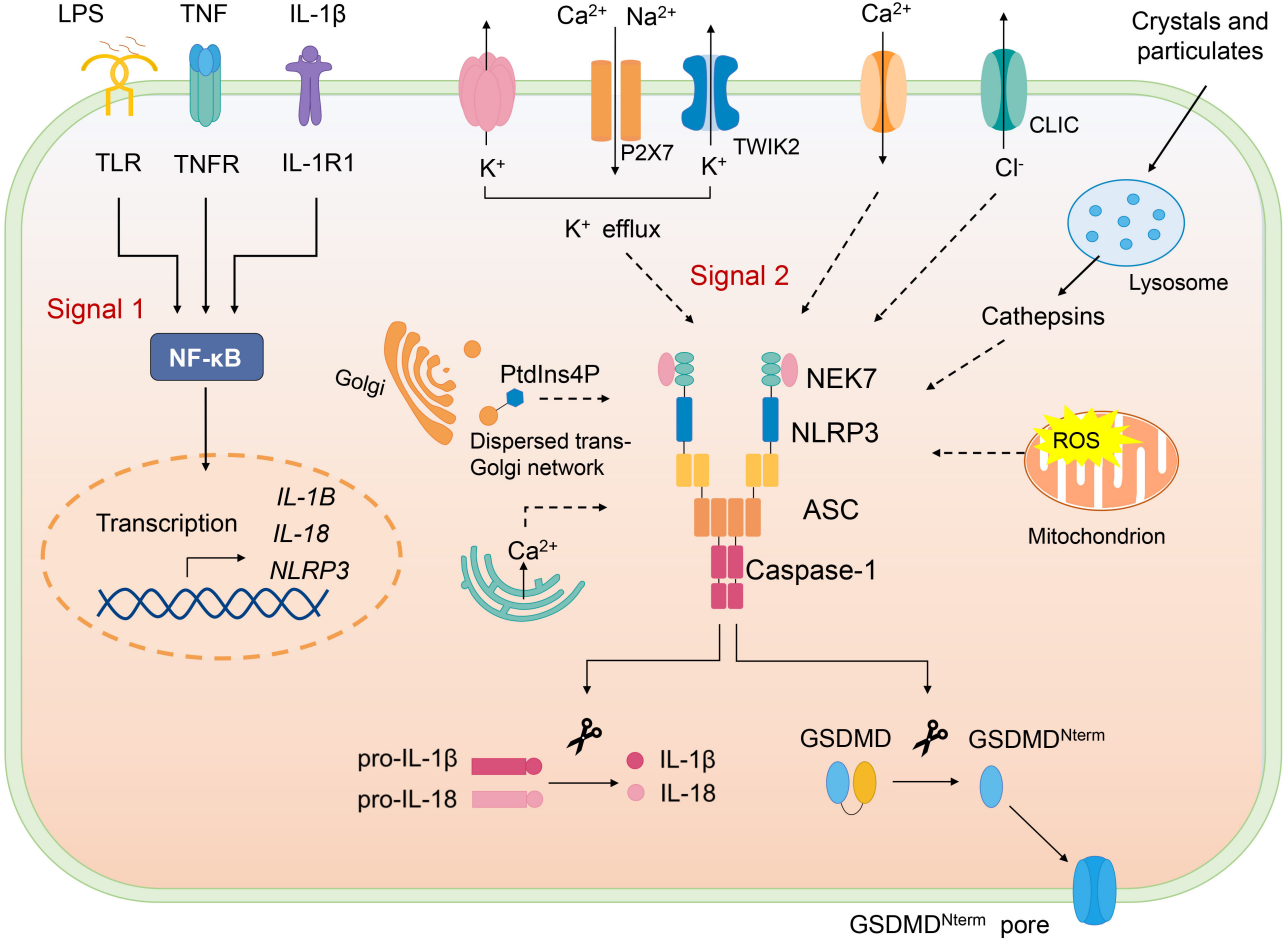
Canonical NLRP3 inflammasome. Canonical NLRP3 inflammasome activation requires two signals: The signal 1 (priming) involves PAMPs or cytokines-induced NF-κB activation and upregulates the gene expression of NLRP3 and the proinflammatory cytokines. Signal 2 is provided by various PAMPs and DAMPs; they activate multiple upstream signaling events, such as K^+^ efflux, Ca^2+^ flux, Cl^-^ efflux, mtROS production, release of oxidized mitochondrial DNA, lysosome damage, and Golgi disassembly (dispersed trans-Golgi network). NLRP3 inflammasome assembly triggers the auto-cleavage of caspase-1, which in turn cleaves pro–IL-1β and pro–IL-18. Active caspase-1 also cleaves GSDMD and releases GSDMD-N from the autoinhibition and free GSDMD-N oligomerizes in membranes to form pores and induce pyroptosis.

Canonical NLRP3 inflammasome activation requires two signals, priming and activation (**Figure 2**). NLRP3 inflammasome can be primed with various pathogen-associated molecular patterns (PAMPs) to activate NF-κB to induce transcription of NLRP3 and the key proinflammatory cytokines such as pro–IL-1β. Priming signal also mediates multiple post-translational modifications (PTMs) of NLRP3, which licenses NLRP3 for subsequent activation. For example, LPS priming downregulates FBXL2-induced ubiquitination and degradation of NLRP3 and increases its life span. LPS exposure induces level of an E3 ligase FBXO3, which targets FBXL2; the latter recognizes Trp-73 within NLRP3 for interaction and targets Lys-689 (human) for ubiquitin ligation and degradation (24). Moreover, priming triggers phosphorylation of NLRP3 at S194 (mouse) by JNK-1, which facilitates deubiquitylation of NLRP3 and its self-association (25).

Following the priming step, NLRP3 is activated by a second signal and initiates its self-oligomerization, activated NLRP3 forms inflammasome together with ASC and pro–caspase-1, causing full activation of NLRP3 inflammasome, which is defined as the “activation step”. Various stimuli, namely, PAMPs and DAMPs can offer the activation signal for NLRP3 inflammasome. To date, several upstream signals, which are interrelated and overlapping, have been proven to trigger assembly of NLRP3 inflammasome.

K^+^ efflux is considered as an important upstream signal of NLRP3 inflammasome activation. At first, potassium-proton ionophore nigericin and ATP were reported to cause the decrease of intracellular K^+^, which is indispensable for release of IL-1β, and K^+^ channel blockers inhibit the processing of IL-1β (26–28). It has recently been reported that P2X7-mediated cations influx promotes K^+^ efflux through TWIK2 upon ATP stimulation (29). Apart from several NLRP3 activators (such as nigericin) that can permeate the cell membrane to K^+^, particulate matter can also trigger K^+^ efflux, highlighting the significant role of K^+^ efflux in NLRP3 inflammasome activation (30). Nonetheless, NLRP3 activation could also be induced in K^+^ efflux-independent way. Imiquimod and CL097, the small molecules targeting mitochondria trigger ROS, induce NLRP3 activation independently of K^+^ efflux (31).

Ca^2+^ mobilization has been reported to be crucial for NLRP3 activation (32, 33). Upon NLRP3 activation stimulated by various agonists(nigericin, ATP, alum, and monosodium urate [MSU]), Ca^2+^ is mobilized. Blocking Ca^2+^ mobilization impairs NLRP3 inflammasome assembly and activation (33). However, how Ca^2+^ mobilization activates NLRP3 remains not clear. It is suggested that excessive ER release of Ca^2+^ leads to mitochondrial Ca^2+^ overload, which, in turn, causes mtROS production, an important trigger of NLRP3 inflammasome activation (34–36). Moreover, inhibitors of Ca^2+^ mobilization block mtROS production during NLRP3 inflammasome activation by ATP (33). However, a study documents that increased cytosolic Ca^2+^ induced by Ca^2+^ ionophore or Ca^2+^-mobilizing GPCR is not sufficient to activate NLRP3 inflammasome (37).

An increase of extracellular Cl^-^ has been demonstrated to inhibit IL-1β release (38, 39), chloride intracellular channel proteins CLIC1 and CLIC4 deficiency impairs pro-IL-1β transcription and IL-1β secretion (40). Further study shows CLICs act downstream of K^+^ efflux-mtROS axis, mtROS induces CLICs translocation to plasma membrane and the subsequent chloride efflux, promoting NEK7-NLRP3 interaction, which is essential for inflammasome assembly (41). These researches suggest that chloride efflux is an important for NLRP3 activation. In addition, Cl^-^ efflux has been reported to induce ASC oligomerization but that is inactive in the absence K^+^ efflux, which is necessary for NLRP3-NEK7 interaction and caspase-1 cleavage (42).

Lysosomal disruption is crucial for crystal-induced NLRP3 inflammasome activation. Multiple crystals could activate NLRP3, including inhaled silica crystals (43), uric acid (44), cholesterol (9), and fibrillar peptide amyloid-β (45). NLRP3 activation by crystals requires phagocytosis of crystals, which results in lysosomal damage and rupture. In a study, sterile lysosomal rupture caused by Leu-Leu-OMe (L-leucyl-L-leucine methyl ester) is sufficient to trigger NLRP3 inflammasome activation, whereas suppression of phagosomal acidification or cathepsin B (using Ca074Me as the inhibitor) blocks NLRP3 activation (43). However, Ca074Me is reported to inhibit multiple cathepsins, and NLRP3 inflammasome activation is mediated by several redundant cathepsins (including cathepsin B) (46).

Mitochondrion dysfunction is also involved in NLRP3 inflammasome activation. Inside the cell, damaged organelles are cleared by autophagy; autophagic proteins deficiency leads to the increased number of dysfunctional mitochondria and mitochondrial DNA (mtDNA) release, thus activating NLRP3 inflammasome (47). Mitochondrion ROS could also trigger NLRP3 activation, blocking key enzymes of the respiratory chain with rotenone or antimycin leads to mtROS production and then activates NLRP3, and inhibition of autophagy/mitophagy also results in accumulation of mtROS and NLRP3 activation (47). AIM2 inflammasome is also responsible for dsDNA recognition; however, oxidized mtDNA preferentially triggers NLRP3 not AIM2 inflammasome (48). CMPK2 is an enzyme that supplies deoxyribonucleotides for mtDNA synthesis, TLR signals *via* Myd88 and TRIF induce IRF1-dependent transcription of CAMK2, and CAMK2-mediated mtDNA synthesis is required for oxidized mtDNA generation induced by NLRP3 agonists (49). Moreover, mitochondrial lipid cardiolipin was found to interact with NLRP3 directly and induce its activation, suggesting that cardiolipin may serve as a mitochondrial docking site for NLRP3 as well as activating ligand (50). Nevertheless, cardiolipin participates in multiple mitochondrion processes involved in NLRP3 inflammasome activation, such as mitophagy, mitochondrion fission, and fusion (51), so whether cardiolipin directly activates NLRP3 remains further study.

Recently, the role of disassembly of the trans-Golgi network (TGN) in NLRP3 inflammasome activation has been revealed (52). Upon stimulation by NLRP3 agonists, TGN is dissembled into vesicles (dispersed TGN, dTGN), phosphatidylinositol-4-phosphate (PtdIns4P) (negatively charged) on the dTGN forms ion bond with the polybasic region of NLRP3, recruiting NLRP3 to dTGN, where NLRP3 aggregates into puncta, resulting in oligomerization of ASC and subsequent caspase-1 activation. The translocation of NLRP3 to dTGN is crucial for both K^+^ efflux dependent and independent inflammasome activation (52). During K^+^ efflux–dependent NLRP3 inflammasome activation, K^+^ efflux is demonstrated to be required for the recruitment of NLRP3 to TGN, possibly promoting ionic binding *via* lowering cellular ionic strength, but the subsequent activation does not require K^+^ efflux. Although stimulated by imiquimod and CL097(K^+^ efflux-independent stimuli), the recruitment is barely affected by extracellular KCl (52).

NLRP3 inflammasome activation also induces pyroptosis, characterized by pore formation in the plasma membrane, causing membrane rupture and release of cytosolic contents. Gasdermin D (GSDMD) is reported to be the executor of pyroptosis induced by NLRP3 inflammasome agonists (53, 54). GSDMD-N domain possesses the pyroptosis-inducing activity and is inhibited by its C domain (GSDMD-C), upon inflammasome activation, caspase-1 cleaves GSDMD into two parts, the free GSDMD-N oligomerizes in membranes to form pores, thus killing cells from inside (55, 56). *In vitro*, GSDMD-N is found to kill cell-free bacteria, implying its direct bactericidal effect but further study is needed (56). Moreover, it is demonstrated that GSDMD mediates IL-1β release from living macrophages under the hyperactivation state, GSDMD pores on the membrane facilitate and acts as a conduit for IL-1β secretion (57).

### 2.2 Non-canonical inflammasome activation

**Figure 3:**
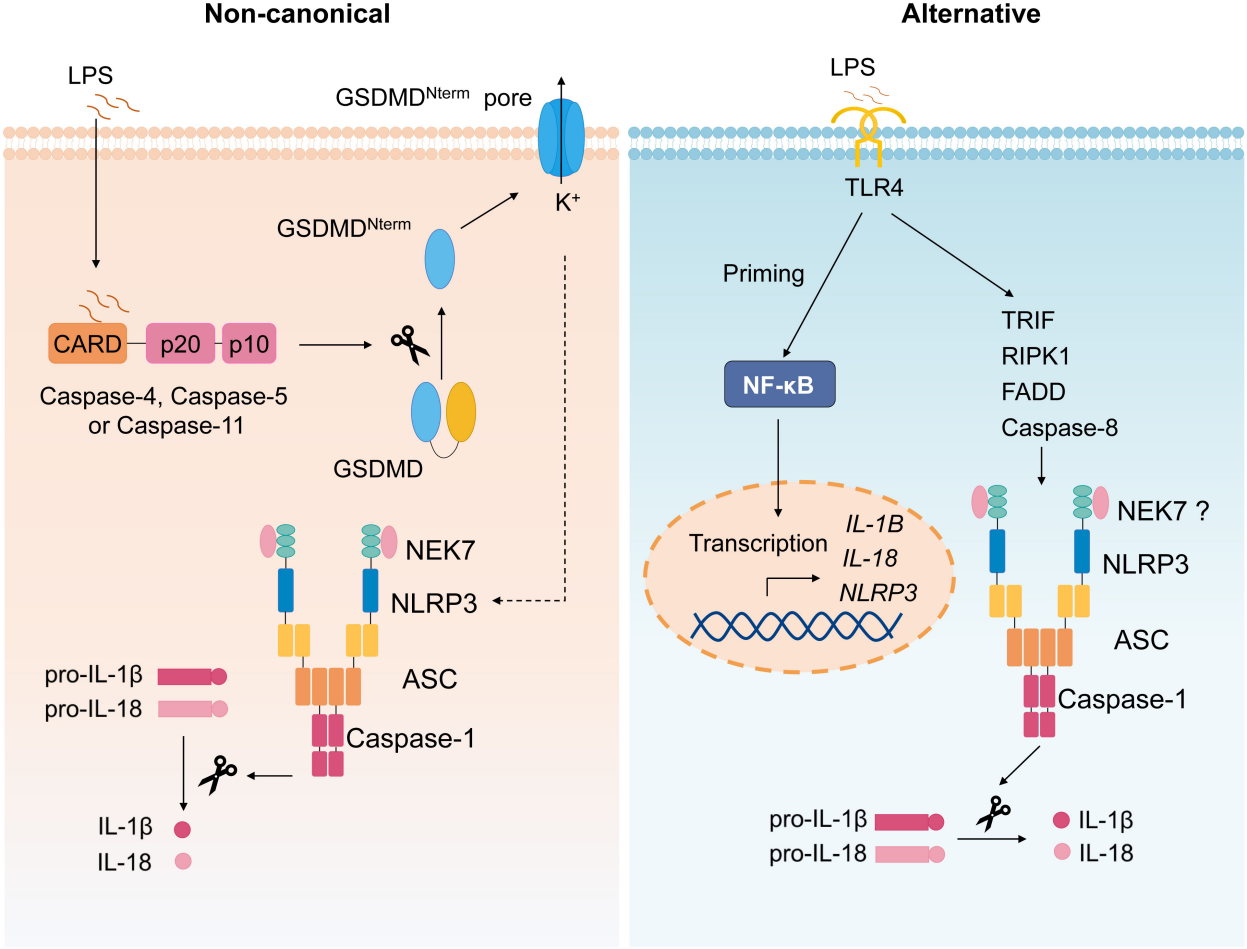
Non-canonical and alternative inflammasome. Non-canonical inflammasome activation is triggered by caspase-4/5/11 activation upon recognition of intracellular LPS, followed by cleavage of GSDMD and subsequent pyroptosis, causing the potassium efflux, which then induces canonical NLRP3 inflammasome. The alternative NLRP3 inflammasome activation refers to the signal 1 only inflammasome response. In monocytes, TLR ligands alone trigger IL-1β secretion *via* TLR4-TRIF-RIPK1-FADD-CASP8 signaling upstream of NLRP3.

The non-canonical inflammasome refers to the caspase-11–dependent inflammasome (**Figure 3**) (58). During various bacterial infection, cytoplasmic LPS is recognized by caspase-11, which then mediates non-canonical inflammasome activation (59, 60); thus, caspase-11 plays a critical role in septic shock (59–61). It is demonstrated that mouse caspase-11 (human caspase-4 and caspase-5) directly binds to intracellular LPS followed by its oligomerization and activation (62). Upon activation, Caspase-4/5/11 also cleaves GSDMD and induces pyroptosis (53, 54), the active caspase-11 causes potassium efflux to induce canonical NLRP3 inflammasome activation, leading to IL-1β secretion (63). Oxidized phospholipids (oxPAPC), one class of DAMPs, can bind to caspase-11 and induce inflammasome activation, but oxPAPC only induces caspase-11–dependent IL-1 release, but not pyroptosis (64).

### 2.3 Alternative inflammasome activation

Alternative NLRP3 inflammasome activation refers to the signal 1 only inflammasome response (**Figure 3**) (65). Initially, it is reported that TLR ligands alone could stimulate human monocytes to secrete IL-1β (66, 67). Moritz M. Gaidt et al. demonstrated that, in human and porcine monocytes, alternative inflammasome activation was mediated by TLR4-TRIF-RIPK1-FADD-CASP8 signaling upstream of NLRP3. Unlike canonical inflammasome, alternative response does not induce pyroptosome formation or pyroptosis (65). Apart from human and porcine monocytes, murine DCs are also reported to secrete mature IL-1β stimulated by TLRs agonists, which was mediated by NLRP3 inflammasome but independent on the purinergic P2X7 receptor (68).

## 3 NLRP3 and diseases

Due to the significant role of NLRP3 inflammasome in inflammatory responses, its activation must be tightly controlled. Aberrant NLRP3 inflammasome activation is considered to be responsible for the autoimmune disease CAPS, caused by the gain of function mutant of NLRP3. Many chronic diseases are driven by inflammatory responses in the presence of danger signals formed in pathology situations; these danger signals could stimulate NLRP3 inflammasome to induce secretion of proinflammatory cytokines including IL-1β and IL-18, promoting disease progression.

Gain-of-function mutation of NLRP3 has been documented to trigger CAPS (69–71). The CAPS refers to a class of autoinflammatory disorder with cutaneous, ocular, musculoskeletal, and neurologic disease symptoms combined with chronic inflammation. To date, more than 200 mutations of NLRP3 associated with CAPS have been reported in the INFEVER website (72), almost all the mutations lie in the NACHT domain. In CAPS patients, gain-of-function mutation of NLRP3 leads to the aberrant inflammasome activation and increased IL-1β secretion, resulting in the systemic inflammation and disease systems. Thus, the anti–IL-1β therapy is recommended for CAPS (73). To date, three anti–IL-1β therapies are available including the recombinant form of IL-1RA (Anakinra) (74), the anti-IL-1β monoclonal antibody (Canakinumab) (75), and the dimeric IL-1 receptor fusion protein (Rilonacept) (76).

The main characteristic of atherosclerosis is the plaques in arteries, which is made up of fat, cholesterol, calcium, or other substances in the blood. Recent studies have demonstrated that atherosclerosis is a chronic inflammatory disease; various anti-inflammatory agents are tested in clinical trials to treat atherosclerosis (77, 78). In carotid atherosclerosis plaques, the expression of NLRP3 inflammasome components is elevated, implying the association of NLRP3 with atherosclerosis (79). Furthermore, it has been reported that cholesterol crystal, a hallmark of atherosclerotic lesions (80), activates NLRP3 inflammasome in a process involving lysosomal rupture, and reconstitution with NLRP3-, ASC-, or IL-1α/β–deficient bone marrow in low-density lipoprotein receptor (LDLR)–deficient mice shows reduced early atherosclerosis, suggesting the link between NLRP3 inflammasome and pathogenesis of atherosclerosis (9). Additionally, oxidized LDL (oxLDL) triggers NLRP3 inflammasome activation *via* CD36-meidated unabated uptake of oxLDL, leading to its transformation into intracellular cholesterol crystals that activate NLRP3. Upon response to oxLDL, CD36 also cooperates with TLR4-TLR6 to activate the priming signal to induce expression of NLRP3 and pro–IL-1β (81). Taken together, DAMPs during AS initiate both priming signal and activation signal to induce NLRP3 inflammasome activation, promoting the pathogenesis of AS.

NALFD (non-alcoholic fatty liver disease) is strongly related with metabolic syndrome; its prevalence is increasing around the world. Non-alcoholic steatohepatitis (NASH) is the progressive subtype of NAFLD, with steatosis, hepatocyte injury (ballooning), and inflammation (82). Although often asymptomatic, NASH can progress to cirrhosis. So far, no medicines have been approved to treat NAFLD or NASH. During the last few years, increasing evidences illustrate the role of NLRP3 in NASH (83–85), elevated hepatic expression of Nlrp3, Asc, and Casp1 associates with liver inflammation in dietary and nutrient deficiency fatty liver diseases (84, 86–88), and NLRP3 activation is critical for progression of NASH evidenced by genetic knockout mice and NLRP3 pharmacological inhibitor (12, 89, 90).

Emerging evidences have shown the essential role of chronic inflammation in type 2 diabetes (T2D) (91). IL-1β is reported to inhibit insulin signaling (92–94); randomized clinical trials shows that blocking IL-1β signaling (using recombinant human IL-1 receptor antagonist) is effective in type 2 diabetes (94). With the discovery that NLRP3 inflammasome mediates caspase-1 activation and IL-1β maturation, its relation with insulin resistance and type 2 diabetes development has been recognized (13, 95–97); NLRP3 deficiency protects mice from obesity-induced insulin resistance (13, 96). Moreover, the level of plasma free fatty acids (FFAs) are elevated in T2D patients and diabetic animals (98); FFAs could activate NLRP3 inflammasome activation through AMPK-ROS signaling axis, possibly elucidating the mechanism by which HFD induces inflammatory response, which promotes insulin resistance (97).

Gout is an inflammatory arthritis associated with precipitation of MSU within the joint (99, 100). IL-1β have been implicated as an important proinflammatory cytokine in gout, inducing the influx of neutrophil into the synovium and joint fluid (101). The link between MSU crystal and IL-1β production remained unknown until Martinon et al. reported that MSU stimulation-activated NLRP3 inflammasome, macrophages deficient in NLRP3, and ASC or caspase-1 were defective in IL-1β secretion in response to MSU, deficiency of ASC, or caspase-1 impaired the neutrophil influx in an animal model of MSU-induced peritonitis (11). Similar results are demonstrated with the causal agent of pseudogout, calcium pyrophosphate dihydrate (CPPD) (11). Therefore, NLRP3 inflammasome is demonstrated to link causal agents of gout and the disease pathogenesis.

Neurotic plaques and neurofibrillary tangles (NFTs) in the brain are hallmarks of (AD), formed by accumulation of amyloid-beta (Aβ) and hyperphosphorylated tau respectively, together leading to neurodegeneration and cognitive decline (102). Aβ activates NLRP3 in a process engaging the lysosomal damage and cathepsin B release (45); cleaved caspase-1 is increased in amyloid-plaque in mice and patients with AD (10, 45). In a model of AD, deficiency of ASC or NLRP3 alleviates amyloid plaque pathology (103), implying that NLRP3 is critical in the progression of amyloid-beta pathology. Moreover, a recent study demonstrates the pivotal role NLRP3 in tau pathology; NLRP3 can be activated by tau monomers and oligomers, then inducing tau hyperphosphorylation and aggregation possibly *via* regulation of tau kinase (104). α-synuclein, the abnormal aggregation of which is the main pathogenesis of Parkinson’s disease (PD), also activates NLRP3 inflammasome. In patients with PD and PD model mice, abnormal NLRP3 inflammasome activation has been observed; inhibition of NLRP3 inflammasome effectively ameliorated dopaminergic degeneration and pathological α-synuclein accumulation in mice (105). Thus, NLRP3­mediated neuro­inflammation could be an important driver of PD progression.

Drug-Induced Liver Injury (DILI) is the main reason hindering drug development and for drug withdrawal. There is a growing number of evidences linking NLRP3 inflammasome activation with DILI induced by multiple drugs (**Table 1**). N-acetyl-p-aminophenol (acetaminophen, APAP) is a commonly used antipyretic and analgesic drug. APAP is safe at normal dose but causes sever liver failure and even death when taken in excess (115). Imaeda and colleagues demonstrated that deficiencies in NLRP3 inflammasome components ameliorated APAP-induced liver injury and death; anti–IL-1β antibody also exhibited protective effect in mice administered with a lethal dose of acetaminophen (106). Additionally, NLRP3 inflammasome activation is implicated in liver injury induced by antituberculosis Drugs (Rifampicin [RIF] and Isoniazid [INH]), NLRP3 inhibitor (INF39 or CP-456773) ameliorated liver injury caused by Isoniazid and Rifampicin (107). Moreover, INH has been reported to induce NLRP3 inflammasome activation in a sirtuin1 (SIRT1)–dependent manner (108). NLRP3 inflammasome activation was also observed in mice treated with Triptolide (TP, a main ingredient of Chinese Traditional Medicine, Tripterygium wilfordii Hook. f), which causes hepatotoxicity. Moreover, caspase-1 inhibitor pretreatment effectively alleviated the TP-induced liver toxicity (110).

**Table 1:** The role of NLRP3 inflammasome in Drug-Induced Liver Injury (DILI).

Name	Structure	The role of NLRP3 inflammasome in DILI	Refs
APAP	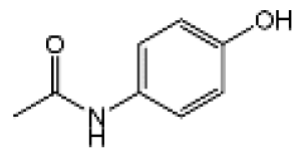	Deficiencies in NLRP3 inflammasome components and administration of anti-IL-1β antibody alleviated APAP-induced death and liver injury in mice	(106)
Rifampicin	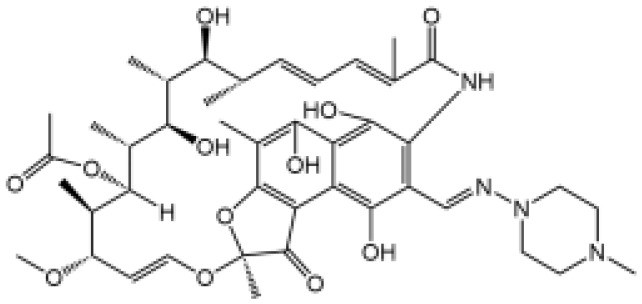	Combination of Isoniazid and Rifampicin induced NLRP3 inflammasome activation *in vivo*, NLRP3 inhibitor (INF39 or CP-456773) ameliorated liver injury induced by Isoniazid and Rifampicin	(107)
Isoniazid	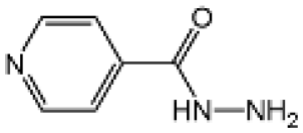	NLRP3 inhibitor (INF39 or CP-456773) ameliorated liver injury caused by Isoniazid and Rifampicin. Isoniazid activated NLRP3 inflammasome in SIRT1-dependent manner	(107, 108)
Carbamazepine	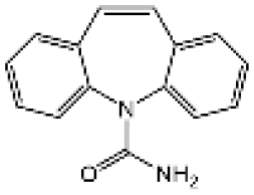	CBZ promoted NLRP3 inflammasome activation induced by ATP or nigericin *via* increasing mtROS production. NLRP3 deficiency in mice protects from the severe liver damage caused by cotreatment of CBZ and LPS.	(109)
Triptolide	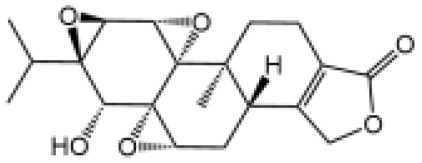	Mice treated with Triptolide (TP) exhibited liver injury along with activation of NLRP3 inflammasome, caspase-1 inhibitor effectively alleviated the TP-induced liver toxicity	(110)
Icariside II	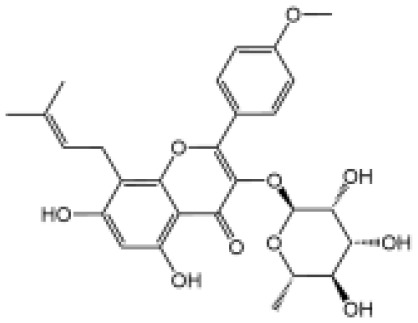	Icariside II enhanced ATP or nigericin-induced NLRP3 inflammasome activation *via* promotion of mtROS production, a combination of non-hepatotoxic doses of LPS and ICS II caused liver injury, which is alleviated by Nlrp3 deficiency or administration of MCC950 (a specific NLRP3 inhibitor)	(111)
Icariside I	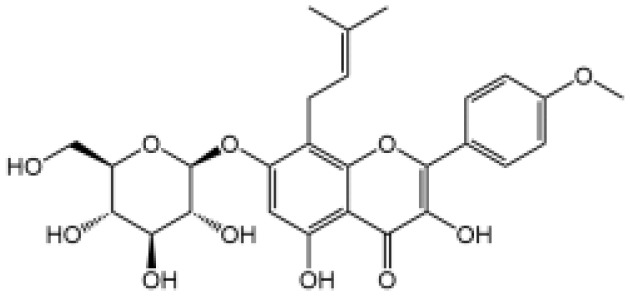	Icariside I enhanced NLRP3 inflammasome activation induced by ATP or nigericin *via* increasing the production of mtROS, MCC950 amelioreated liver injury caused by combination of non-hepatotoxic doses of LPS and Icariside I	(112)
Bavachin	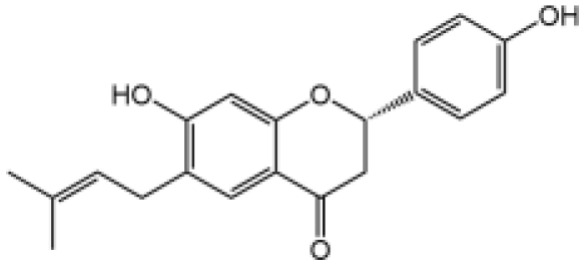	Bavachin boosted ATP or nigericin-induced NLRP3 inflammasome activation *via* increasing mtROS production, MCC950 suppressed liver injury caused by LPS/bavachin	(113)
Psoralidin	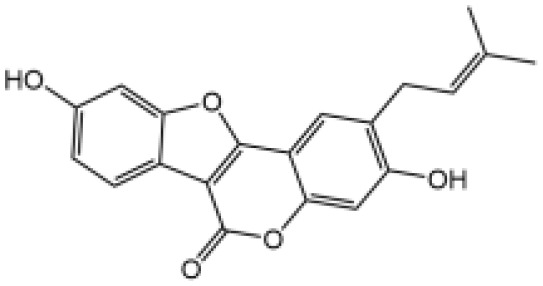	Psoralidin induced liver injury in a susceptible mouse model of lipopolysaccharide (LPS)-mediated IDILI. Psoralidin induced caspase-1 cleavage and secretion of IL-1β in BMDMs, MCC950 treatment partially attenuated that	(114)

In our studies, we found the widely used antiepileptic agent carbamazepine (CBZ), which causes idiosyncratic liver injury (116) and enhances NLRP3 inflammasome activation through promotion of mtROS production; *in vivo* data showed that cotreatment of CBZ and LPS caused severe liver damage in WT mice but not in NLRP3^−/−^ mice, suggesting the significance of NLRP3 activation in CBZ-induced liver injury (109). Meanwhile, we have also reported that several components of *Epimedii Folium* and *Psoraleae Fructus*, which are traditional Chinese medicine that have been suggested to induce liver injury (117, 118), could enhance NLRP3 activation and caused liver injury in a LPS-mediated susceptibility mouse model of IDILI. These components include icariside II (111), icariside I (112), and bavachin (113). These three components promote NLRP3 activation stimulated by ATP or nigericin *via* increasing the production of mtROS. Liver injury caused by these compounds could be alleviated by NLRP3 deficiency or MCC950 administration (111–114). Psoralidin is a component of *Psoraleae Fructus*, induced liver injury in a susceptible mouse model of LPS-mediated IDILI. Psoralidin alone could induce caspase-1 cleavage and IL-1β secretion in BMDMs, but MCC950 only partially attenuated that (114), suggesting that psoralidin activates NLRP3 inflammasome as well as other inflammasomes.

## 4 Pharmacological inhibitors of NLRP3 inflammasome

**Figure 4:**
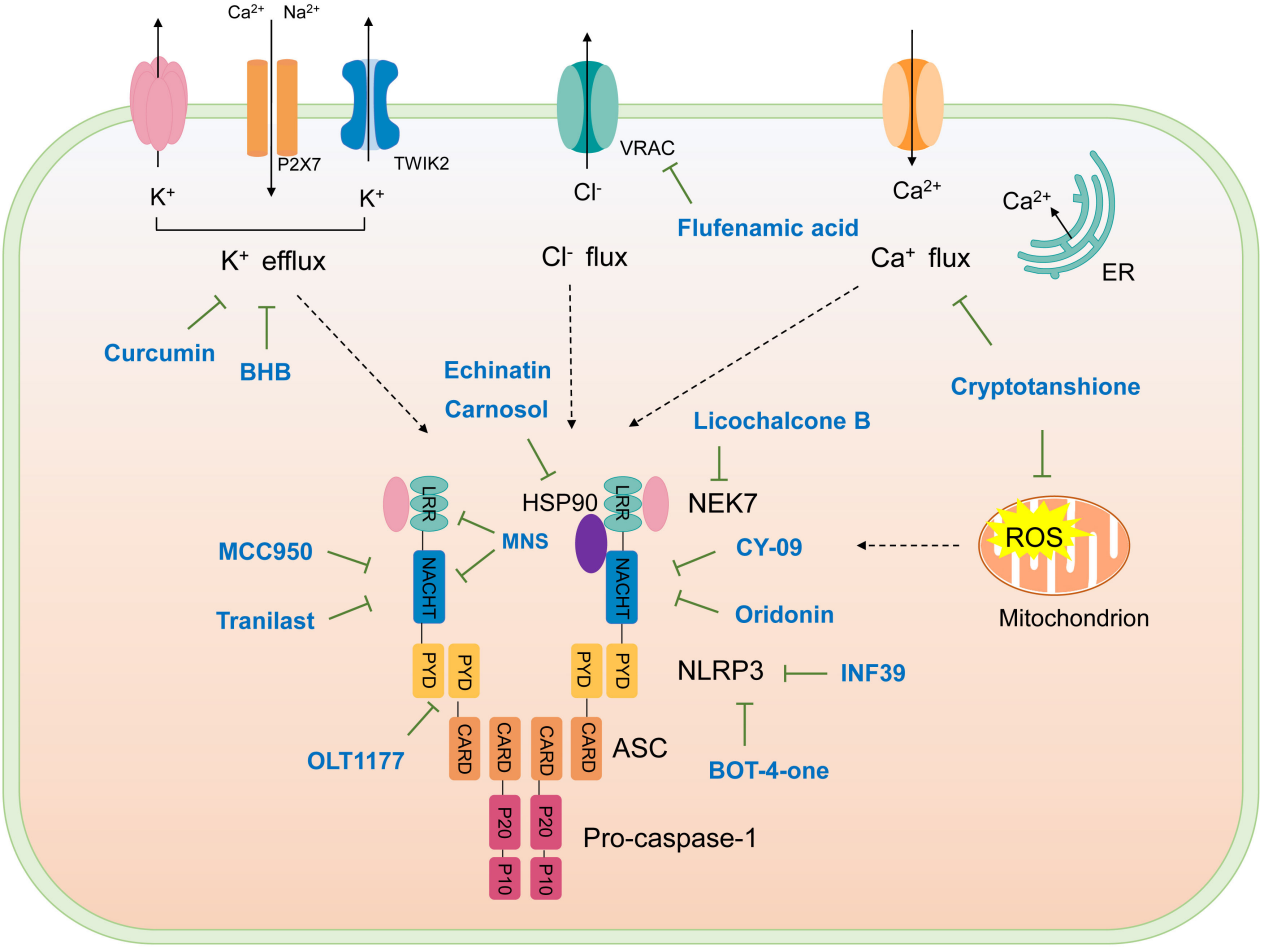
Schematic illustration of targets of NLRP3 inflammasome inhibitors. Upon activation, NLRP3 oligomerizes through interactions between NACHT domains, then recruiting ASC *via* PYD-PYD interactions, assembled ASC recruits caspase-1, forming NLRP3 inflammasome. NKE7 binds to NLRP3; the interaction is necessary for NLRP3 inflammasome assembly. HSP90 is suggested to bind NLRP3 to promote its activation. K^+^ efflux, Ca^2+^ flux, Cl^-^ efflux, and mtROS production are upstream signaling events of NLRP3 activation. The targets of NLRP3 inflammasome inhibitors are shown.

The studies revealing the crucial role of NLRP3 in various inflammatory diseases demonstrate that pharmacological intervention aimed at this critical target may hold therapeutic promise. Although IL-1β–targeted therapies have been used in clinic to treat the related inflammatory disease, NLRP3 inflammasome-targeted therapies have a competitive advantage. Recently, more and more inhibitors of NLRP3 inflammasome have been identified (**Figure 4** and **Table 2**).

**Table 2:** Inhibitors of NLRP3 inflammasome.

Inhibitor	Structure	Mechanism of action	Inhibition of priming	Refs
Glyburide	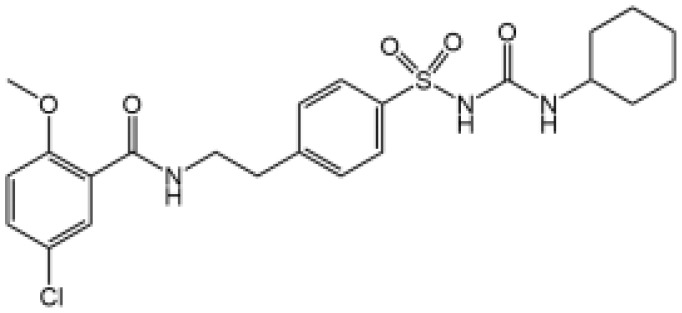	N/A	N/A	(119–122)
MCC950	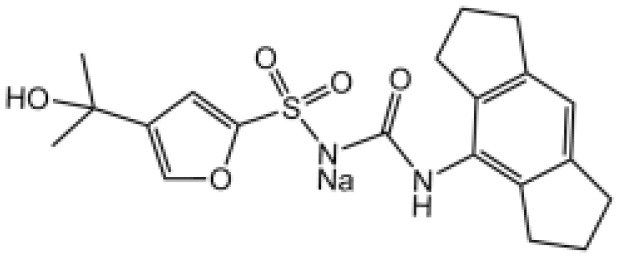	Binds to walker B motif of NATCH domain to inhibit ATPase activity and close active conformation	No	(123–126)
Flufenamic acid (Fenamate)	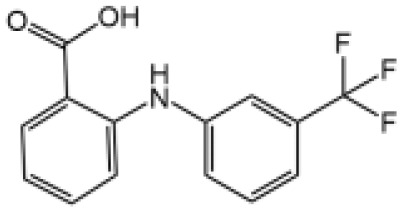	Reversible blockade of Cl channel volume-regulated anion channels (VRAC)	N/A	(127)
CY-09	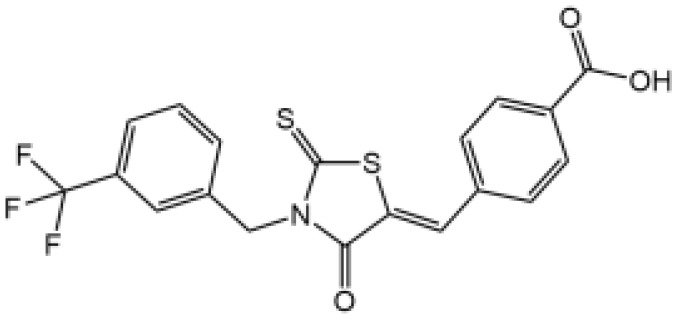	Binds to the Walker A motif of NLRP3 to impair the ATP binding of NLRP3 and suppresses its ATPase activity	No	(128)
Tranilast	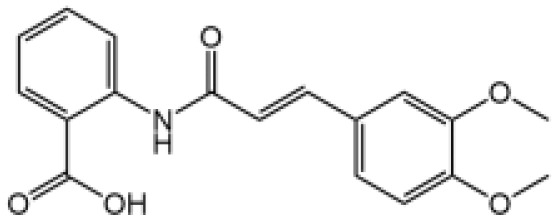	Binds to the NACHT domain of NLRP3 to block NLRP3 oligomerization	Yes	(129)
MNS	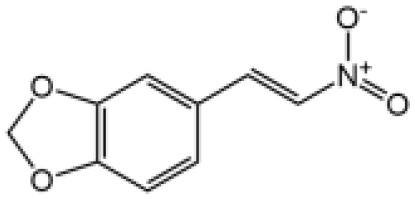	Binds with NACHT and LRR domain and inhibit ATPase	Yes	(130)
Bay11-7082	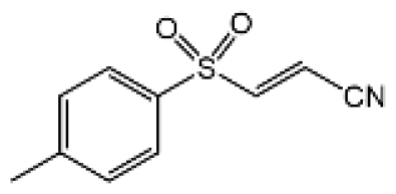	Inhibits ATPase activity of NLRP3	Yes	(131)
OLT1177	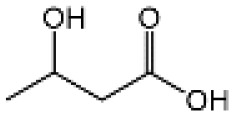	Prevents NLRP3-ASC and NLRP3-caspase-1 interaction, inhibits ATPase activity of NLRP3	No	(132)
BHB	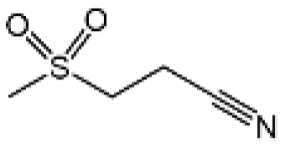	Prevents K^+^ efflux	N/A	(133)
INF39	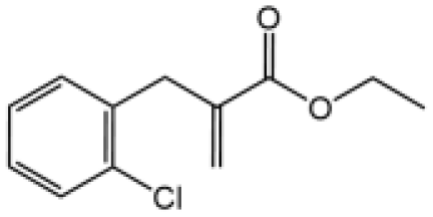	Irreversibly interacts with NLRP3 and affects NLRP3 conformational change that could be related with the ATPase activity	Yes	(134)
BOT-4-one	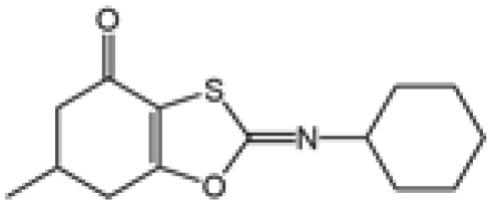	NLRP3 alkylation, impairs ATPase activity of NLRP3, enhances the ubiquitination level of NLRP3	Yes	(135)
Oridonin	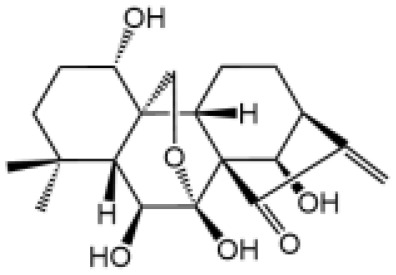	Covalently bond with the cysteine 279 of NLRP3 in NACHT domain to inhibit NLRP3-NEK7 interaction	No	(136)
Licochalcone B	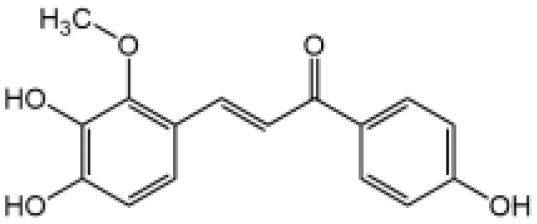	Binds to NEK7 and disrupts NEK7-NLRP3 interaction	Yes	(137)
Echinatin	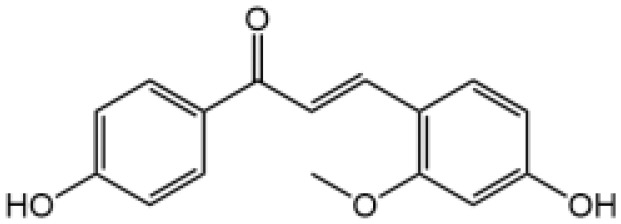	Binds to HSP90, blocks its ATPase activity and abolishes the association between the cochaperone SGT1 and HSP90-NLRP3	N/A	(138)
Curcumin	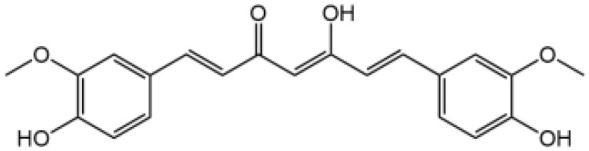	Prevents K^+^ efflux, inhibits microtubule-driven recruitment of ASC on mitochondrion to NLRP3 on the endoplasmic reticulum	Yes	(139)
Cardamonin	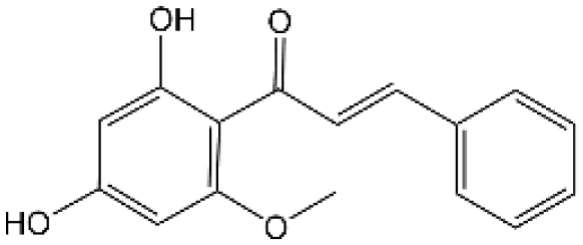	N/A	No	(140)
Carnosol	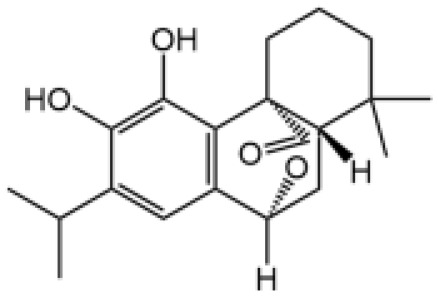	Binds to HSP90 and inhibits HSP90 activity	Yes	(141)
Cryptotanshinone	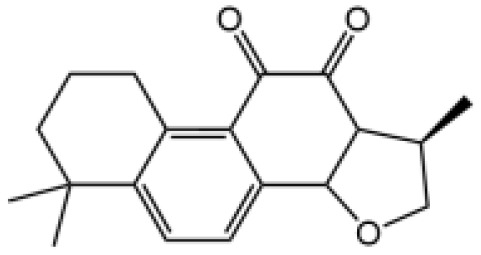	Suppresses Ca^2+^ signaling and mitochondrial reactive oxygen species (mtROS) induction	No	(142)

N/A, Not Applicable.

MCC950, also known as CP-456,773, is the best characterized inhibitor of NLRP3. Initially, MCC950 was reported as a potent IL-1β processing inhibitor *via* screening of a group of diarylsulfonylurea­containing compounds (124), then Coll and colleagues demonstrate that MCC950 specifically inhibits NLRP3 activation and ASC oligomerization, without affecting K^+^ efflux, Ca^2+^ flux or NLRP3–ASC interactions (125). It remains unclear how MCC950 inhibits NLRP3 activation until two recent studies report that MCC950 directly binds to NLRP3 Walker B site within the NACHT domain, inhibiting its ability to hydrolyze ATP and closing the active conformation of NLRP3 (123, 126). MCC950 was even tested to treat rheumatoid arthritis in phase II clinical trials but failed because of the increase of serum liver enzyme levels, which are liver toxicity signals (77).

OLT1177 is a β-sulfonyl nitrile that specifically blocks NLRP3 inflammasome activation. Nanomolar concentrations of OLT1177 suppressed NLRP3 inflammasome activation *in vitro* but did not impair AIM2 or NLRC4 inflammasome (132). OLT1177 not only prevents NLRP3-ASC and NLRP3-caspase-1 interaction but it also blocks NLRP3 ATPase activity; however, OLT1177 does not affect pro-IL-1β gene expression. In humans receiving OLT1177 at oral doses up to 1000 mg daily for 8 days, no adverse effects or hematological or biochemical changes were observed, showing the safety of OLT117 in human (132). OLT1177 is now tested in clinic to treat acute gout flares and heart failure (78).

Tranilast (TR) is an analog of a tryptophan metabolite used in clinic to treat inflammatory diseases (143). TR also has inhibitory effect on cytokine-induced NF-κB activation (144). TR impaired pro–IL-1β expression induced by LPS in BMDMs, but when BMDMs were stimulated with TR for 30 min after LPS treatment, TR did not affect NLRP3 and pro–IL-1β expression but still inhibited NLRP3 inflammasome activation (129). TR bound to NLRP3 at the NACHT domain and blocked its oligomerization, but it did not affect its ATPase activity. TR exhibited beneficial effects in gouty arthritis, CAPS and type 2 diabetes in mouse models (129).

CY-09 is an analog of CFTR(inh)-172 (C172) without cystic fibrosis transmembrane conductance regulator (CFTR)–inhibitory activity (145), C172 was identified as an inhibitor of NLRP3 from screening a compound library, and as an analog of C172, CY-09 was tested and showed obviously inhibitory effect on NLRP3 activation (128). CY-09 directly binds to NLRP3 at the Walker A motif in NACHT domain, impairing ATP binding of NLRP3 and inhibiting its ATPase activity. Moreover, CY-09 exhibits therapeutic effect on mice model of MSU-induced peritonitis (128).

Several other compounds have been demonstrated to target NLRP3 and inhibit its ATPase activity, such as Bay11-7082, MNS(3,4-methylenedioxy-β-nitrostyrene), IFN39, and BOT-4-one. Bay11-7082 is known as NF-κB inhibitor to block the IKKβ activity; it has been reported to abrogate caspase-1 activation *via* affecting LPS priming (146, 147). Christine Juliana and co-workers discovered that Bay11-7082 could block NLRP3 activation independently on its effect on NF-κB, through impairing NLRP3 ATPase activity (131). MNS is a reported inhibitor of Syk kinase, but its inhibitory effect on NLRP3 inflammasome is independent on Syk. MNS binds to NLRP3 at the NOD and LRR domain, blocking its ATPase activity possibly through cysteine modification (130). INF39 was identified through the screening of acrylate derivatives designed to inhibit NLRP3, INF39 interrupts NLRP3 activation through direct irreversible binding to NLRP3 and blocking its ATPase activity (134). BOT-4-one is a novel benzoxathiole derivative that has been reported as NF-κB inhibitor through targeting IKKβ (135). BOT-4-one directly targets NLRP3 *via* alkylation to suppress its ATPase activity, it also enhances the ubiquitination level of NLRP3 (135). However, the safety and protective role of these compounds in NLRP3-driven diseases have not been investigated thoroughly.

Glyburide is a widely used drug approved by FDA to treat type 2 diabetes (148), working by inhibiting ATP-sensitive K^+^ (K_ATP_) channels in pancreatic β cells (149). It is the first identified compound to inhibit NLRP3 inflammasome activation, but the effect is independent on KATP channels or ATP-binding cassette transporters (119), which was proposed as the glyburide target (121). Glyburide acts upstream of NLRP3 to block IL-1β secretion induced by various stimuli (119), but the direct target needs further study. Several studies report that Glyburide reduces a variety of NLRP3-dependent pathologies such as septic shock, bronchopulmonary dysplasia and Cutaneous leishmaniasis (119, 120, 122).

NSAIDs are characterized by COX inhibitors (150) and approved to treat multiple diseases. Michael J.D. Daniels and colleagues demonstrate Flufenamic acid and mefenamic acid, which belong to the fenamate class of NSAIDs, specifically block NLPR3 inflammasome activation through reversible blockade of Cl^-^ channel volume-regulated anion channels (VRAC), independently of the effect on COX (127). Mefenamic acid also exhibited protective effects in a rodent model of Amyloid beta-induced memory loss and 3× TgAD transgenic mice (a mouse model for AD) (127). The reversible inhibitory effect of fenamate NSAIDs on NLRP3 offers significant clinical benefit, flufenamic acid and mefenamic acid are readily approved by FDA to be used in clinic, they can be quickly repurposed as drugs to treat AD or maybe other NLRP3-driven diseases.

BHB is a type of ketone bodies from the liver of mammals, it serves as alternative energy source during states of energy deficit (151). BHB specifically inhibits NLRP3 inflammasome independently of starvation-regulated mechanisms, but mediated by blockade of potassium efflux and ASC oligomerization (133). The protective role of BHB in NLRP3-driven diseases such as Muckle–Wells syndrome, urate crystal–induced peritonitis, and familial cold autoinflammatory syndrome has been validated in rodent models (133). As a naturally occurring metabolite, BHB has great potential to be developed as a safe drug to treat NLRP3-mediated diseases.

Many Chinese medicinal herbs exhibit obviously anti-inflammatory effects and have been used to treat various inflammatory diseases. Recently, our group and others’ have identified various inhibitors of NLRP3 inflammasome from traditional Chinses medicinal herbs

Oridonin (Ori) is the active component of *Rabdosia rubescens*, which is a commonly used traditional Chinese medicinal herb to treat inflammatory diseases (152). Ori specifically blocks NLRP3 inflammasome activation, without affecting LPS-induced priming (136). Ori covalently binds to Cys279 of NLRP3 *via* its carbon-carbon double-bond and block NEK7-NLRP3 interaction, thus blocking subsequent NLRP3 inflammasome assembly (136). Although there is possibility that covalent drugs may exhibit idiosyncratic toxicity or hypersensitivity, they have pharmacological advantages such as enhanced efficacy and prolonged duration of action (153, 154). Thus, Ori may have a significant potential to be used in NLRP3-driven inflammatory diseases.

Licochalcone B is the main component of the traditional Chinese herbal medicine Glycyrrhiza plants (licorice). Our study demonstrates that Licochalcone B exhibits specifically inhibitory effect on NLRP3 inflammasome but does not affect potassium efflux, mtROS production or calcium flux (137). Licochalcone B binds to NEK7 and blocks the interaction between NEK7 and NLRP3. Licochalcone B has been reported to affect NF-κB pathway (155), when administered before LPS priming, it slightly suppresses pro–IL-1β production (137). The protective effect of Licochalcone B has been investigated in mice models of septic shock, NASH, and peritonitis. No changes in biochemical parameters reflecting liver and kidney function were observed in mice treated with 40mg/kg of Licochalcone B for 34 days, suggesting its safety (137).

Echinatin is also the bioactive component of the traditional Chinese herbal medicine Glycyrrhiza plants (licorice). We have identified that Echinatin potently restrains NLRP3 inflammasome activation *via* affecting the association between SGT1 and HSP90-NLRP3 (138). HSP90 and its client-adaptor SGT1 are essential for NLRP3 activity (156), echinatin directly binds to HSP90 and inhibits its ATPase activity, causing the dissociation of SGT1 from HSP90 and NLRP3, thereby blocking subsequent inflammasome assembly and activation (138). Moreover, HSP90 and SGT1 also play a critical role in NLRC4 activation (156), consistently, echinatin also impairs NLRC4 activation (138) and may also be therapeutic in NLRC4-mediated diseases.

Curcumin is a polyphenolic compound of the rhizomes of turmeric (157), which has been widely used in foods as spice and coloring and traditional medicine. Curcumin plays a beneficial role in various inflammatory diseases including diabetes, rheumatoid arthritis and cardiovascular diseases (158). Curcumin showed specific inhibition for NLRP3, but not for AIM2 or NLRC4 inflammasome (139). Importantly, curcumin blocks potassium efflux and disturbs the mitochondrial transport as well as ASC polymerization, thus preventing NLRP3 inflammasome assembly. Curcumin also ameliorated MSU-induced peritoneal inflammation and HFD-induced insulin resistance through inhibiting NLRP3 inflammasome activation in mice models (139).

Cardamonin is an active ingredient of traditional Chinese medicinal herb *Alpinia katsumadai*; it has been reported to alleviate inflammatory bowel disease by the inhibition of NLRP3 inflammasome activation (159). One of our recent studies demonstrates that cardamonin specifically inhibits NLRP3 inflammasome activation, it has no effect on the priming stage, but blocks NLRP3-dependent ASC oligomerization (140). In a mouse model, cardamonin obviously ameliorates LPS-induced septic shock and IL-1β production (140).

Carnosol, a natural polyphenol, was first isolated from sage (*Salvia carnosa*) *(* 160). Carnosol was identified to inhibit NLRP3 inflammasome activation through a high-throughput assay for caspase-1 activity to screen NLRP3 inhibitors (141). Carnosol blocks NLRP3 activation *via* binding to HSP90 and inhibiting its ATPase activity. The therapeutic effects of carnosol on LPS-induced septic shock and MCD -induced NASH have been verified in mouse models (141). Mice administered with carnosol (120 mg/kg) i.p. for two weeks showed no change in body weight or biochemical parameters of liver and kidney function (141), indicating the potential of carnosol to be a safe candidate for the development of therapeutic drug for NLRP3-driven diseases.

Liu et al. reported cryptotanshinone as a potent inhibitor of NLRP3 activation (142). Cryptotanshinone is a main component of the traditional medicinal herb *Salvia miltiorrhiza* Bunge, it has been reported to suppress NF-κB activation and reduce inflammation-induced by LPS (161). Cryptotanshinone could block NLRP3 inflammasome activation, the inhibitory effect was independent on its regulation of NF-κB pathway (142) and limited to NLRP3, not affecting AIM2 or NLRC4 inflammasome activation. Moreover, cryptotanshinone impairs calcium flux and the induction of mtROS, it demonstrated significantly preventive property in mice models of septic shock and MCD-induced NASH (142). Further studies about the exact target of cryptotanshinone are awaited.

## 5 Conclusion and perspectives

NLRP3 inflammasome is central to immune responses triggered by danger signals derived from both pathogens or host. With the intensive studies on its mechanism of activation, much attention has been paid to the relation between aberrant NLRP3 activation and the pathogenesis of various diseases, including those related with metabolic disorder and aging. Currently, the number of individuals affected by the inflammatory conditions is growing rapidly as a result of the improvement of living standards and aging population, there is an increasing demand for NLRP3 inflammasome targeted therapeutics.

The current treatment for NLRP3-mediated diseases is mainly by targeting IL-1β, but IL-1β maturation is also mediated by activation of other types of inflammasomes; blockade of IL-1β may affect the host defense response. Therapeutics targeting NLRP3 inflammasome is more specific, with better efficacy and safety. At present, many NLRP3 inhibitors have been reported; among them, MCC950 is the best studied with high specificity. Many derivatives based on MCC950 are under development, and several derivatives of MCC950 have also entered clinical phase II. In addition, another NLRP3 inhibitor OLT1177 is now tested in clinic for the treatment of acute gout flares and heart failure. With the in-depth understanding of the high-resolution structure and activation mechanism of NLRP3, it will be more conducive to the development of NLRP3 targeted drugs.

Meanwhile, various inhibitors of NLRP3 inflammasome from traditional Chinese medicinal herbs have been reported, many traditional Chinese medicinal herbs have been used in the treatment of inflammatory diseases and show good safety, so it may hold great promise for screening safe and effective inhibitors for NLRP3 inflammasome from traditional Chinese medicinal herbs, which may be developed as candidate therapeutic drugs for treating NLRP3-mediated diseases.

## Author contributions

XZ wrote the manuscript, XZ and QL drew the figure and collected the tables. GX contributed to language modification and content adjustment. ZB and XX supervised and revised the manuscript. All authors approved the submitted version.
